# Model building and assessment of the impact of covariates for disease prevalence mapping in low-resource settings: to explain and to predict

**DOI:** 10.1098/rsif.2021.0104

**Published:** 2021-06-02

**Authors:** Emanuele Giorgi, Claudio Fronterrè, Peter M. Macharia, Victor A. Alegana, Robert W. Snow, Peter J. Diggle

**Affiliations:** ^1^ CHICAS, Lancaster Medical School, Lancaster University, Lancaster, UK; ^2^ Population Health Unit, Kenya Medical Research Institute-Wellcome Trust Research Programme, Nairobi, Kenya; ^3^ Centre for Tropical Medicine and Global Health, University of Oxford, Oxford, UK

**Keywords:** disease mapping, explanatory modelling, geostatistics, predictive modelling, prevalence, spatial correlation

## Abstract

This paper provides statistical guidance on the development and application of model-based geostatistical methods for disease prevalence mapping. We illustrate the different stages of the analysis, from exploratory analysis to spatial prediction of prevalence, through a case study on malaria mapping in Tanzania. Throughout the paper, we distinguish between predictive modelling, whose main focus is on maximizing the predictive accuracy of the model, and explanatory modelling, where greater emphasis is placed on understanding the relationships between the health outcome and risk factors. We demonstrate that these two paradigms can result in different modelling choices. We also propose a simple approach for detecting over-fitting based on inspection of the correlation matrix of the estimators of the regression coefficients. To enhance the interpretability of geostatistical models, we introduce the concept of domain effects in order to assist variable selection and model validation. The statistical ideas and principles illustrated here in the specific context of disease prevalence mapping are more widely applicable to any regression model for the analysis of epidemiological outcomes but are particularly relevant to geostatistical models, for which the separation between fixed and random effects can be ambiguous.

## 1. Introduction

In this paper, our aim is to provide a guiding framework for the formulation, application and validation of geostatistical models [1] for disease prevalence mapping. Model-based geostiatistics (MBG) has been extensively used to address scientific problems whose primary objective is to make probabilistic inference on a spatially continuous phenomenon, using data collected over a finite set of geo-referenced locations.

Here, we focus on cross-sectional surveys that are conducted in order to understand the spatial variation of disease prevalence within a geographical area of interest. In low-resource settings, disease registries are typically absent and cross-sectional surveys often provide the only source of health outcome data that can be used to infer the disease burden in a population. In this context, the available data consist of a set of household locations *x*_*i*_, the numbers *n*_*i*_ of individuals who have been tested for the disease of interest, and the numbers *y*_*i*_ out of *n*_*i*_ who have returned a positive test result. MBG provides a statistically principled, likelihood-based approach for predicting disease prevalence, *p*(*x*), at any desired location *x* in the area of interest by exploiting the spatial correlation between the observations *y*_*i*_. In order to improve the efficiency of spatial predictions for prevalence, MBG models also allow the inclusion of covariates, *d*(*x*), i.e. variables that are observed and recorded at a location *x*, and are considered to be associated with *p*(*x*). In this paper, our focus will be on the use of covariates *d*(*x*) that are available as a regular grid of locations covering the study area (also known as *raster data*) and are used as a proxy for the distribution of disease vectors or to capture socio-economic inequalities in the population. In the context of mosquito-borne diseases in developing countries, two examples of this kind of covariate are vegetation density, which quantifies the suitability of a location to serve as a mosquito habitat, and night-time light (NTL), which is often used as a proxy for the local level of economic development. Because of their high-spatial resolution, raster data are especially useful for prediction of *p*(*x*) at locations where no data have been collected. However, this leads to two fundamental questions. How should we model the relationship between *d*(*x*) and *p*(*x*) in MBG models? And how should we assess the impact of *d*(*x*) on the spatial predictions of *p*(*x*)?

The answers to those questions might differ according to the scientific objective of the study. As in Shmueli [2], we distinguish between *explanatory modelling* and *predictive modelling*. Under explanatory modelling the model-building process, and in particular the selection of candidate covariates, should be informed by context-specific scientific knowledge of the underlying disease process, to the extent that this is well understood. Also, interest lies principally in understanding how risk-factors represented by the covariates *d*(*x*) relate to disease prevalence *p*(*x*). In predictive modelling, although context-specific scientific knowledge can still play an important role, the effort is directed primarily towards the development of a model that can predict as accurately as possible future data generated by the same underlying process. For these reasons, different concerns arise under the two paradigms. For example, in explanatory modelling accounting for measurement error in the covariates is important in order to obtain unbiased estimates of regression parameters that link *d*(*x*) to *p*(*x*). In predictive modelling, the selection of covariates is carried out in order to minimize a measure of prediction error and considerations about how accurately a regression parameter *d*(*x*) measures the effect of a putative risk factor are of less concern.

Current applications of disease prevalence mapping in low-resource settings have adopted different approaches to the development and application of geostatistical models, especially in relation to the selection and use of covariates for spatial prediction. The field of malaria epidemiology offers many interesting examples that illustrate this. Weiss *et al.* [3] propose an algorithmic approach to select a large number of covariates for mapping malaria prevalence across Africa, using standard, non-spatial logistic regression. Specifically, starting with an initial set of more than 50 million covariates including transformations and interactions of well-established malaria risk factors, the authors proceed through five steps of model selection by making extensive use of cross-validation and the Akaike information criterion for ranking different models. After reducing the number of covariates to 1887, a sixth step is carried out to identify a final set of 20 covariates, each of which is randomly drawn from the remaining set by giving preference to those showing better predictive performance.

In a more recent paper, Bhatt *et al.* [4], predict malaria prevalence using 15 pre-selected covariates without any further reduction. In a first stage, a predefined set of regression and non-parametric models are fitted separately to the data in order to capture complex interactions between covariates and their nonlinear relationships with prevalence. The resulting predictions for prevalence from each of the applied methods are then combined, using an approach that they term *Gaussian process stacked generalization*. This delivers a single estimate which is then re-used as a predictor in a linear geostatistical model for the logit-transformed prevalence. Using cross-validation, Bhatt *et al.* [4] show that this approach outperforms other interpolation methods, including standard geostatistical models where covariates are introduced simply as regression terms in the linear predictor for prevalence. Here, the development of the model-fitting algorithm is exclusively focused on reducing a specified index of predictive performance in a hold-out sample. However, the improved predictive performance is obtained at the expense of the interpretability and, hence, the explanatory power of the model.

Within a Bayesian inferential setting, a widely used approach to variables selection for malaria prevalence mapping has been inspired by the seminal paper of George & Mulloch [5] in which a latent binary variable is introduced into the model in order to identify subsets of the most important covariates. Diboulo *et al.* [6] use this approach in the development of a geostatistical model for mapping malaria in Burkina Faso. Each of the regression coefficients associated with a covariate is given *a prior* consisting of a mixture of two zero-mean Gaussian distributions with the variance of one of the two constrained to be 1000 times smaller than the variance of the other. The mixing probability of the two distributions is assumed to be 0.5 *a priori* and a covariate is then retained in a subsequent fit of the model if there is a posterior probability larger than 0.5 that favours the Gaussian distribution with the larger variance. Variations of this approach have also been used by Giardina *et al.* [7], Adigun *et al.* [8] and, for schistosomiasis prevalence mapping, Chammartin *et al.* [9]. One of the main advantages of these approaches is that the variable selection process takes into account the spatial correlation in the data, unlike in Weiss *et al.* [3]. However, several arbitrary choices are made in the specification of the mixture distributions and it is unclear to what extent these affect the identification of important covariates.

By contrast, Macharia *et al.* [10] and Giorgi *et al.* [11] fit spatio-temporal geostatistical models to malaria prevalence data in Kenya and Somalia without using any covariates other than the age of the examined individuals at each sampled location. The authors’ rationale for this choice was a concern for the misspecification of the regression relationship between *d*(*x*) and *p*(*x*), which might yield invalid inferences for *p*(*x*) in areas where, due to the absence of data, these would be entirely driven by the covariates *d*(*x*). However, the use of an MBG model without any spatially referenced covariates questions the face validity of predictions that consequently revert to the mean prevalence level at prediction locations remote from the sampled locations. Also, it is unclear to what extent misspecifications of regressions relationships between covariates and prevalence might distort the resulting predictions. We investigate both of these issues in the present paper.

A variety of approaches that have been used for building geostatistical models in the context of other tropical diseases include Slater *et al.* [12] and Moraga *et al.* [13] for lymphatic filariasis; Zourè *et al.* [14] and O’Hanlon *et al.* [15] for onchocerchiasis; Magalhães *et al.* [16] and Lai *et al.* [17] for soil-transmitted helminths.

Through a geostatistical analysis of malaria prevalence data in Tanzania, this paper describes how to develop geostatistical models, from the first stage of exploratory analysis to the final step of spatial prediction. Simulation studies are also carried out in order to offer further insights into the use and misuse of covariates for disease prevalence mapping. The data and R scripts for the exploratory analysis, parameter estimation and spatial prediction are freely available at: github.com/giorgilancs/covariates.

## 2. Introducing the worked example

Throughout the paper, we use the data from the Tanzania Demographic and Health Survey and Malaria Indicators Survey conducted in 2015 (henceforth, DHS-MIS2015) [18]. This consists of 387 geo-referenced locations representing *clusters* of households falling within predefined geographical areas, known as census enumeration areas (EAs), which were delineated for the 2012 Tanzania Population and Housing Census.

The survey employed a two-stage stratified sampling design to provide estimates for the entire country, for urban and rural areas in Tanzania Mainland, and for Zanzibar. For specific indicators, the sample design allowed the estimation of indicators for each of the 30 regions (25 regions from Tanzania Mainland and five regions from Zanzibar) while the rest were representative for each of the nine zones. Stratification was achieved by separating each region into urban and rural areas, resulting in 59 sampling strata. In the first stage, clusters consisting of EAs were selected from the 2012 Tanzania Population and Housing Census sampling frame. In the second stage, 22 households were systematically selected from each cluster from a complete listing of households within each selected cluster. In all selected households, with the parent’s or guardian’s consent, children age 6–59 months were tested for *Plasmodium falciparum*.

The research question we address is: in what areas of Tanzania is malaria prevalence above 0.3? As in previous studies [10,19], here, we use a 0.3 (i.e. 30%) prevalence threshold to define high malaria burden areas that require intensive and sustained vector control.

Our outcome variable is the number of positive test results, *y*_*i*_, based on a rapid diagnostic test (RDT) for *Plasmodium falciparum* antigenaemia, out of *n*_*i*_ examined individuals at location *x*_*i*_, for *i* = 1, …, *N* with *N* = 790.

In our analysis, we consider the following spatially referenced candidate covariates for modelling *P. falciparum*, all of which have been used in most previous studies on malaria risk mapping [20–22].

— *Population density*, obtained from the WorldPop database (www.worldpop.org), is used to account for the higher levels of malaria transmission in low-populated rural areas. This is available as a raster file at a resolution of 3 arc (approx. 100 m at the equator).
— *NTL* captures the level of urbanization of a location [23] and is complementary to population density. As a result of lower poverty rates and increased access to health facilities, malaria risk is indeed lower in urban areas [24,25]. A 2013 gridded surface of intercalibrated NTL was obtained from the Geodata portal (geodata.globalhealthapp.net) at a spatial resolution of 1 km^2^.
— *Rainfall* is an important environmental factor that directly affects the suitability of potential breeding sites for Anopheles mosquitoes. Here, we use the annual mean precipitation based on the Climate Hazards Group InfraRed Precipitation with Station data (CHIRPS) [26] at a 5 × 5 km spatial resolution.
— *Temperature* is known to affect the survival and development of *P. falciparum* from larvae into viable adults. In this analysis, we consider annual mean temperatures for 2015 at 5.6 × 5.6 km spatial resolution from Moderate-resolution Imaging Spectroradiometer (MODIS) sensor was available at the Land Processes Distributed Active Archive (LP DAAC) Center (lpdaac.usgs.gov/products/mod11c1v006/).
— *Enhanced vegetation index* (EVI) acts as a proxy for the presence of suitable mosquitoes breeding sites. Unlike other indices of vegetation (e.g. the normalized difference vegetation index), EVI corrects for some distortions in the reflected light caused by the particles in the air as well as the ground cover below the vegetation. Here, we use EVI imagery data available at approximately 0.25 × 0.25 km spatial resolution from MODIS.

The different steps of a geostatistical analysis are summarized in table 1. In what follows, each section of the paper corresponds to a different step presented in table 1. We consider steps 1 to 4 to be an essential component of any geostatistical analysis. Step 5 is specifically applicable to the primary objective of this paper, i.e. the assessment of the impact of covariates for prevalence mapping.

**Table 1.** Summary of the steps carried in a geostatistical analysis, highlighting its objectives and the statistical tools used to pursue these. Note that the list of statistical tools presented in the table is not exhaustive and is limited to those presented in this paper.

step	objectives	statistical tools
1. Exploratory analysis	1a. Explore the relationship between prevalence and covariates.	1a. Linear splines regression analysis of each covariate on logit-transformed prevalence.
	1b. Assess the evidence of residual spatial correlation.	1b. Empirical variogram and permutation test for spatial independence.
2. Geostatistical model formulation and parameter estimation	2a. Specify model assumptions on the spatial correlation and non-structured over-dispersion.	2a. Inclusion of a spatial Gaussian process and/or unstructured random effects.
	2b. Obtain parameter estimates and measures of uncertainty.	2b. Likelihood function (or posterior density, if Bayesian inference is used).
	2c. Assess overfitting due to an exceedingly large number of covariates and/or to an over-complex specification of the nonlinear regression relationship.	2c. Correlation matrix of the regression parameter estimates.
3. Spatial prediction	3a. Define the predictive target and their spatial scale (e.g. regional average prevalence).	
	3b. Carry out predictions for the pre-defined predictive targets and identify areas of exceedingly high or low risk, depending on the study objective.	3b. Exceedance probabilities.
4. Model validation	4a. Assess the compatibility of the chosen correlation function.	4a. Empirical variogram.
	4b. Assess the calibration of the fitted geostatistical model.	4b. Probability integral transform.
	4c. Assess the assumption of conditional independence.	4c. Empirical variogram and permutation test for spatial independence.
5. Assessment of the contribution of covariates to spatial prediction	5. Estimate and carry out predictions for models using subsets of covariates.	5. Theoretical variograms and summaries of predictive performance (equations (7.3) and (7.4)).

## 3. Exploratory analysis

An initial exploratory analysis of the data provides useful insights into the development of a suitable geostatistical model for prevalence. Here, we focus on two main aspects of exploratory analysis: (i) assessment of the relationship between prevalence and covariates; (ii) testing for residual spatial correlation.

**Figure 1.**
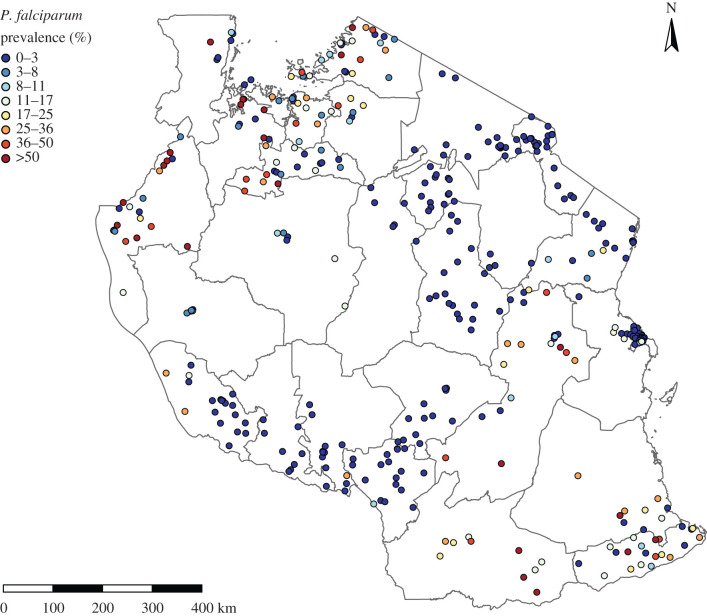
Map of the 387 sampled locations in Tanzania in the year 2015 obtained from Snow *et al.* [27]. The grey lines correspond to the boundaries of the 25 regions of Tanzania.

Figure 1 is a point-map showing the sampled locations of the dataset and their corresponding empirical prevalence. This plot serves two purposes: it visualizes the spatial coverage of the study region; and it gives initial insight into the spatial pattern of prevalence. We observe that the density of the sampled locations corresponds in large part to the distribution of the population in Tanzania, with few or no locations in sparsely populated areas. This feature of the data will be of particular relevance later in the geostatistical analysis, as the sparsity of the data affects both the predictive power of our model and our ability to validate it. In figure 1, we also observe the presence of locations with an empirical prevalence greater than 0.57 in the northwest and in the eastern part of the country. Many locations with no cases reported are found in large swathes of inland Tanzania, especially in the proximity of uninhabited areas.

In order to explore the association of the covariates with prevalence, we use the *empirical logit transformation*, defined as

$$y_{i}^{*}=\log\left(\frac{y_{i}+1/2}{n_{i}-y_{i}+1/2}\right).$$

The rationale for using the empirical logit is that this matches the scale on which covariates are included as terms in the linear predictor of a logistic model; the addition of 1/2 to the numerator and denominator in the equation above is used to deal with *y*_*i*_ = 0 and *y*_*i*_ = *n*_*i*_, for which the standard logit transformation of the empirical prevalence would yield values plus and minus infinity, respectively. We emphasize that the use of the empirical logit is only for exploratory purposes and that the final geostatistical model should instead be based on a binomial likelihood, this being the natural sampling distribution for prevalence data. Fitting a linear geostatistical model to $y_{i}^{*}$ may be a more convenient strategy when *p*(*x*) is close to 0.5 and the *n*_*i*_ are large, but in other circumstances can result in highly biased inferences; see Stanton & Diggle [28] for details.

**Figure 2.**
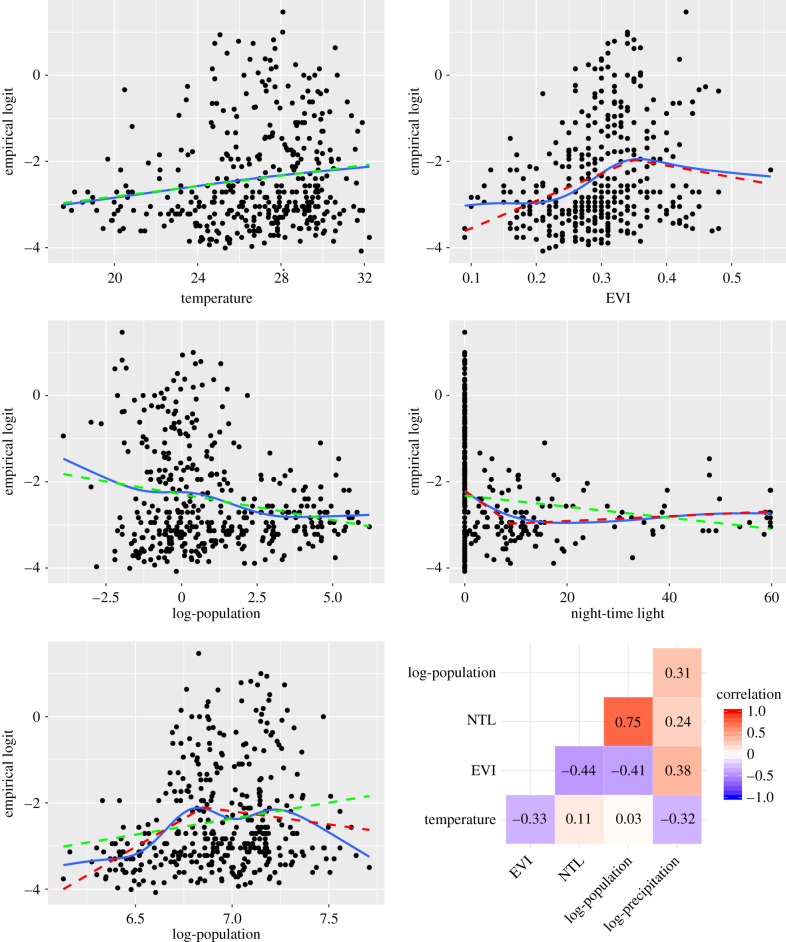
Scatter plots of the empirical logit-transformed prevalence against temperature, enhanced vegetation index (EVI), log-population, night-time light (NTL), log-precipitation and coverage of insecticide treated nets (ITNs). The solid blue and dashed red lines are natural and linear splines, respectively. The dashed green lines for the log-population and log-precipitation are obtained after removing the left knots from the linear splines represented by the red dashed lines. The lower right panel shows the empirical correlation between each pair of covariates.

We next generate scatter plots for $y_{i}^{*}$ against each of the covariates (figure 2). In the case of precipitation and population, we also take the logarithm of the two covariates in order to obtain a more approximately linear relationship with prevalence. We note that all covariates exhibit a very noisy relationship with the $y_{i}^{*}$. Regression splines can be used to obtain a smoothing curve as a better visualization of the nature of any remaining non-linear relationships. In our analysis, we fitted these using the ‘mgcv’ package [29], available in the R software environment. (Its use is illustrated in the R code available at: github.com/giorgilancs/covariates.) The resulting curves (see blue lines of figure 2) provide a useful description of the relationship between the empirical logit and each of the covariates but are not always easy to interpret. To overcome this issue, linear splines, i.e. piece-wise linear functions, also known as broken-stick models, can be used to account for nonlinear relationships with more easily interpretable regression parameters.

In our use of linear splines, we aim to capture two different types of nonlinear relationships: unimodal, where an increasing trend is followed by a decreasing one, or vice versa; or ‘saturation curve’, by which we mean a monotonic relationship that appears to flatten for increasing values of the covariate. Both types of relationships can usually be captured using a linear spline having no more than two knots, say *k*_1_ and *k*_2_, formally expressed in a standard logistic regression as

3.1 $$\begin{array}{rl} \log\left\{\frac{\;p(x_{i})}{1-p(x_{i})}\right\} & =\beta_{0}+\beta_{1}d(x_{i})+\beta_{2}\max\{d(x_{i})-k_{1},0\}\\ & \;+\beta_{3}\max\{d(x_{i})-k_{2},0\}. \end{array}$$

In the above equation, exp{*β*_1_} is the multiplicative effect for a unit increase in *d*(*x*_*i*_) on the odds ratios, for *d*(*x*_*i*_) < *k*_1_. That effect then becomes exp{*β*_1_ + *β*_2_} for *k*_1_ < *d*(*x*_*i*_) < *k*_2_ and exp{*β*_1_ + *β*_2_ + *β*_3_} for *d*(*x*_*i*_) > *k*_2_.

The fitted linear splines are shown in figure 2 by the dashed red lines, whilst the green lines represent simple linear fits. The knots of the splines are chosen through a graphical inspection of figure 2 by placing them in proximity of local maxima of the smoothing spline (blue lines of figure 2). In the case of EVI, NTL and precipitation, the estimated regression relationship, as shown by the red lines, can be described either as a unimodal or a saturation curve, both of which are scientifically interpretable. For example, an increase in the levels of urbanization, as measured by NTL, is associated with a large decrease in prevalence, on average, when moving from rural to moderately urbanized areas. However, for values of NTL larger than 10 the relationship flattens, indicating that higher levels of urbanization are not likely to bring any further decrease in malaria risk. In the case of temperature, values between 25°C and 30°C are considered optimum for *P. falciparum* sporogony [30], which suggests a unimodal effect of temperature on prevalence. The upper panel of figure 2 does not support this and shows instead an approximately linear relationship. The apparent inconsistency may be due to the low number of locations with temperature higher than 30°C. This may have masked any decreasing trend at higher temperatures [31], making any extrapolation based on the green line beyond this range biologically implausible. In the case of the population density covariate, the observed relationship is decreasing and approximately linear which can be explained, similarly to NTL, as a negative association between urbanization and malaria risk.

The next step of the model-building process is to assess if the residual variation, i.e. variation that is not captured by the selected covariates, exhibits evidence of spatial correlation. To this end, we consider a first extension of the standard generalized linear modelling framework in which the notion of residual variation is introduced directly into the linear predictors for the log-odds of prevalence. More specifically, conditionally on a set of independent Gaussian variables *Z*_*i*_, called *random effects*, the count *y*_*i*_ is now assumed to be the realization of a binomial distribution, with linear predictor

3.2 $$\log\left\{\frac{\;p(x_{i})}{1-p(x_{i})}\right\}=d{\left(x_{i}\right)}^{⊤}\beta +Z_{i},$$

where *d*(*x*_*i*_) is a vector of covariates. The model specified in the equation above belongs to the class of generalized linear mixed models and accounts for *overdispersion*, which occurs when the data *y*_*i*_ exhibit greater variation than would be expected under a standard binomial model. After fitting the model, we extract the estimates for the *Z*_*i*_, which we denote by ${\hat{Z}}_{i}$, and calculate their *empirical variogram* to assess whether the ${\hat{Z}}_{i}$ show any evidence of spatial correlation.

Let ${\hat{Z}}_{i}$ and ${\hat{Z}}_{\;j}$ denote the estimates of the random effects associated with locations *x*_*i*_ and *x*_*j*_. The empirical variogram is calculated by averaging the quantities $v_{ij}={\left({\hat{Z}}_{i}-{\hat{Z}}_{\;j}\right)}^{2}/2$ within predefined classes of distance, also known as *bins*. The empirical variogram is then displayed by plotting the averaged *v*_*ij*_ against the mid-points of each distance bin.

Electronic supplementary material 1, figure S2 shows the resulting empirical variogram for the malaria data, after removing the effects of temperature, NTL, population, EVI and precipitation. In the presence of spatial correlation, the typical shape of the empirical variogram is that of a non-decreasing function of distance. This is because spatial correlation would make the squared differences *v*_*ij*_ smaller, on average, if *x*_*i*_ and *x*_*j*_ are closer to each other than if they are distant. In the absence of spatial correlation, the *v*_*ij*_ should exhibit random fluctuations around a constant value. The black line of electronic supplementary material, figure S2 shows a pattern that suggests the presence of spatial correlation. However, this alone does not allow us to conclude that the random effects show evidence of residual spatial correlation; we need to show that the black line is indeed highly incompatible with the absence of spatial correlation. For this purpose, we re-calculate the empirical variogram of the ${\hat{Z}}_{i}$ by randomly allocating the ${\hat{Z}}_{i}$ to the locations *x*_*i*_, and repeat this 10 000 times. The resulting 10 000 variograms can then be used to construct 95% probability intervals for the range of the variation in the *v*_*ij*_ under the assumption of spatial independence. These are denoted in electronic supplementary material, figure S2 by the shaded grey area. The black line falls well outside the 95% envelope, suggesting that the unexplained variation in prevalence by the covariates is spatially correlated.

## 4. Geostatistical model formulation and parameter estimation

In the event that we detect spatial correlation, we further extend the generalized linear mixed model as follows. We now assume that, conditionally on the *Z*_*i*_ and a spatial Gaussian process *S*(*x*_*i*_), the *y*_*i*_ are the realization of a binomial distribution with linear predictor

4.1 $$\log\left\{\frac{\;p(x_{i})}{1-p(x_{i})}\right\}=d{\left(x_{i}\right)}^{⊤}\beta +S(x_{i})+Z_{i}.$$

In this paper, we assume that *S*(*x*) has an exponential covariance function, with variance *σ*^2^. This implies that the distribution of *S*(*x*_*i*_), for *i* = 1, …, *N*, follows a multivariate Gaussian distribution, with correlations expressed by

$$\mathrm{Cor}\{S(x_{i}),S(x_{\;j})\}=\exp\{-u_{ij}/\phi \},$$

where *u*_*ij*_ is the distance between *x*_*i*_ and *x*_*j*_, and *ϕ* is a scale parameter that regulates how quickly the spatial correlation approaches zero for increasing distance *u*_*ij*_. The exponential covariance function corresponds to a mean-square continuous but non-differentiable Gaussian process *S*(*x*). It is a special case of a wider class of covariance functions proposed by Matérn [32] that include an additional parameter to control the mean-square differentiability of the Gaussian process *S*(*x*). This additional parameter is hard to estimate and, in our experience, only materially improves the fit of the model when the empirical variogram shows a clear ‘S’ shape over small distances, which is typical of Matérn covariance functions with *k* > 0.5.

We assume that the *Z*_*i*_ in (4.1) are independent and identically distributed random variables with mean 0 and variance *τ*^2^. The *Z*_*i*_ are called the *nugget effect*, reflecting the historical origin of geostatistical methods in the mining industry. In our context, they can be interpreted as a combination of non-spatial over-dispersion and spatial variation on a scale smaller than the smallest distance between any two data-locations *x*_*i*_. It is impossible to disentangle these two effects without independently replicated data at coincident locations. In this paper, we make the pragmatic choice to exclude *Z*_*i*_ from our spatial predictions of prevalence.

In (4.1), the sum *S*(*x*_*i*_) + *Z*_*i*_ represents all of the variation in prevalence *p*(*x*_*i*_) that is not attributable to the covariates *d*(*x*_*i*_). If all the variables that contribute to the spatial variation in *p*(*x*_*i*_) were available as measured covariates, *S*(*x*_*i*_) would be redundant.

In the geostatistical analysis illustrated in this paper, we estimate the model parameters using the Monte Carlo maximum-likelihood (MCML) method implemented in the PrevMap package [33]. For more technical details, we refer the reader to Geyer’s work [34–36] and to Christensen [37] for an overview of MCML in the context of model-based geostatistics. Here, we only point out that, unlike Bayesian methods of inference, MCML avoids the specification of *prior distributions* for the parameters of the geostatistical model—namely *β*, *σ*^2^, *ϕ* and *τ*^2^—by treating these as unknown constants that are estimated from the data.

Prior distributions are probability distributions that quantify our belief about a model parameter before any empirical evidence is taken into account. In general, choosing a prior is not straightforward. In the recent work by Simpson *et al.* [38] and Fulgstad *et al*. [39], a principled Bayesian framework is developed to define priors that are invariant to reparametrization and penalize for model complexity. However, ideally, a prior should be informed by subject-matter knowledge but translating this into a unique probability distribution can be very difficult; see §4.2.2 of [40] to see some examples. In the absence of prior knowledge, a widely used approach is to define priors with large variances in order to let the data drive the inference on the model parameters. However, with this approach the choice of how large the prior variance should be is often arbitrary. For this reason, in the context of geostatistical analysis where informative priors are rarely available, maximum likelihood is our preferred method of estimation. Against this, an advantage of Bayesian inference is that it naturally incorporates parameter uncertainty into predictions of prevalence within a single, principled probabilistic framework. To overcome this limitation, Giorgi *et al.* [41] propose to use the distribution of the maximum-likelihood estimator as a ‘quasi-posterior’ distribution for the model parameters, and sample from this to propagate parameter uncertainty. When comparing this approach with Bayesian inference and the use of plug-in maximum-likelihood estimates for prevalence prediction, Giorgi *et al.* [41] find only small differences. This is because, in most applications, the predictive variance of the spatial process *S*(*x*) dominates the variance of the parameter estimates.

In order to develop a geostatistical model that is more explicitly informed by knowledge of the disease, it is convenient to group risk factors into different domains, each of which contribute to the transmission of the disease via different routes. In the context of malaria, three main domains are environmental covariates, socio-economic covariates and covariates relating to the history of control interventions. Let $\delta_{\;j}(x_{i})=d_{\;j}{\left(x_{i}\right)}^{⊤}\beta_{\;j}$ denote the cumulative effect on prevalence of the risk factors *d*_*j*_(*x*_*i*_) that fall under the *j*-th domain; we call these *domain effects*. We then re-express model (4.1), as

4.2 $$\log\left\{\frac{\;p(x_{i})}{1-p(x_{i})}\right\}=\sum_{\;j}\delta_{\;j}(x_{i})+S(x_{i})+Z_{i}.$$

Although the model above is mathematically equivalent to that in (4.1), the introduction of the domain effects *δ*_*j*_(*x*_*i*_) can help to improve the interpretability of the model in two different ways. First, at a pre-analysis stage they provide a rationale for the identification of suitable covariates. Second, they guide the variable selection process by requiring each domain to be represented by at least one covariate in the final model used for spatial prediction. In our analysis, the identified covariates provide materially different independent contributions to the explanation of the spatial variation in disease prevalence. For this reason, our approach will be to try to retain as many as possible in the model in order to maximize its explanatory power while guarding against *overfitting*.

Overfitting occurs when the model delivers predictions that follow too closely the observed values of the outcome. Two possible causes of this are (1) the inclusion of too many covariates relative to the sample size and (2) the use of an over-complex regression function to capture nonlinear relationships between the outcome and the covariates. To detect overfitting, the most commonly used approach is *cross-validation*, whereby a randomly selected subset of the data, also referred to as the *training set*, is used to inform predictions at locations of the remainder of the data, or *test set*. Then, based on a prediction error summary, the performance of the model is assessed in both the training and test sets. In case of overfitting of the data, the prediction error obtained from the test set will be substantially larger than that obtained from the train set; for more guidance on how to carry out cross-validation, see §7.10 of [42]. However, this approach has three main limitations: first, it does not provide any insights into the possible causes of overfitting; second, cross-validation becomes less reliable for smaller sample sizes; third, most commonly used prediction error summaries treat the observed fraction of positive cases as the true disease prevalence, which is especially problematic in low prevalence settings.

To overcome these issues when the goal is to carry out variable selection, we propose an alternative approach based on inspection of the correlation matrix of the maximum-likelihood estimates for *β*. The rationale for this is that, in the presence of overfitting, the estimates of the regression coefficients *β* are less stable and tend to become linearly dependent, resulting in correlations close to −1 or 1. This allows us to identify which covariates may be removed from the model and how nonlinear functions, used to model the relationship of the outcome with the covariates, may be simplified.

**Figure 3.**
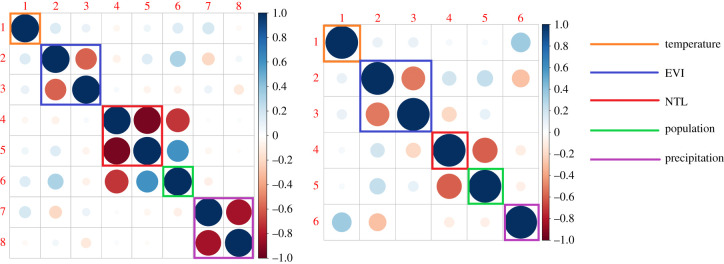
Correlations for the regression parameter estimators. The left panel corresponds to the model that uses the linear splines as indicated by the red dashed lines of figure 2. The right panel corresponds to the model obtained by removing one knot for the population and precipitation covariates, as indicated by the green dashed lines of figure 2.

The left panel of figure 3 shows the correlation matrix for the estimates of *β* based on the linear splines, as represented by the dashed red lines of figure 2. From now on, we shall refer to this initial geostatistical model as $M_{I}$. In this graph, the squares encompass the correlations for all the regression coefficients used to capture the empirical nonlinear relationship between prevalence and a single covariate. We observe that most of the correlations outside of these squares are close to zero, with the exception of two pairs of coefficients for population and NTL whose correlations are approximately −0.7. None of the correlations outside the squares are high enough in absolute value to justify the removal of any of the covariates. If we now examine the correlations of the regression coefficients within each square, we see that, for NTL and precipitation, the estimates have a correlation very close to 1. This strongly suggests overfitting, indicating that the true nonlinear functional relationship may be difficult to recover as a result of the noisy empirical associations, as evident from figure 2. In this case, a possible solution is to fit a simpler linear relationship for NTL and precipitation. In other cases, where more than one knot has been used to define the linear spline, a solution may be the removal of any knot that is close to the minimum or maximum value of an explanatory variable, where nonlinear relationships between the covariates and the outcome are informed by fewer data-points and are therefore less reliable.

We then fit a simplified model where we assume a logit-linear relationship for NTL and log-precipitation. The right panel of figure 3, shows that this has reduced the correlations between the regression coefficients with the largest value around −0.6. We conclude that this simplified model does not show evidence of overfitting. In the next section, we consider spatial prediction based on this simplified geostatistical model, to which we shall refer as $M_{F}$; see table 3 for a summary of the covariates and how these are used in the models $M_{I}$ and $M_{F}$, respectively. The point estimates and 95% confidence intervals for the parameters of $M_{I}$ and $M_{F}$ are reported in electronic supplementary material 1, table S1.

## 5. Spatial prediction

In order to carry out spatial prediction, we first must define (1) a *predictive target*, say T and (2) one or more summary statistics of its predictive distribution, i.e. the probability distribution of T conditional on all of the observed data.

Let *A* denote a geographical area of interest and *R*_*k*_, for *k* = 1, …, *K*, be a set of spatial units forming a partition of *A*. In our example, *A* corresponds to the area encompassed by the boundaries of Tanzania, while *R*_*k*_ are the *K* = 26 Tanzanian regions. In disease prevalence mapping, two predictive targets that are often of scientific interest are the spatially continuous prevalence surface covering the whole of *A*, i.e. *p*(*A*) = {*p*(*x*) : *x* ∈ *A*}, and the regional population prevalence,

5.1 $$p(R_{k})=\frac{\int_{R_{k}}w(x)p(x)\;dx}{\int_{R_{k}}w(x)\;dx},\;k=1,\ldots ,K,$$

where *w*(*x*) is the population density at location *x*. In order to compute the target in the above equation, we approximate each integral by a sum over a regular grid covering *R*_*k*_. In general, the spatial resolution of the regular grid should be fine enough to make the correlation between values of *p*(*x*) in adjacent pixels at least 0.95 in order to generate an appropriately smooth prevalence surface map for *p*(*x*).

The most commonly used summaries of the predictive distribution of a prevalence surface are maps of its means, standard errors and selected quantiles for each spatial unit. However, public health policies are often developed based on the exceedance, or non-exceedance, of predefined prevalence thresholds, say *l*. In these cases, the prediction at each location or region is expressed as an exceedance probability (EP), defined as EP(*x*) = Prob[*p*(*x*) > *l*|*y*_1_, …, *y*_*N*_] and EP(*R*_*k*_) = Prob[*p*(*R*_*k*_) > *l*|*y*_1_, …, *y*_*N*_] for location-specific and spatially aggregated targets, respectively. An EP close to 1 or 0 indicates near-certainty that the target does or does not exceed the specified threshold *l*, whereas an EP close to 0.5 indicates a high degree of uncertainty.

Another important issue is to assess whether spatial predictions at unsampled locations should be conducted outside the empirical ranges of the covariates. Electronic supplementary material 1, S3 shows a 10 by 10 km regular grid covering the whole Tanzania, after removing locations with no inhabitants according to the WorldPop (www.worldpop.org) population density data. Locations in black indicate that the value of at least one of the covariates at that location falls outside the empirical range. In this case, extrapolation should be carried out with caution, especially when the fitted regression relationships may follow trajectories that are not supported by scientific knowledge of the phenomenon under investigation.

**Figure 4.**
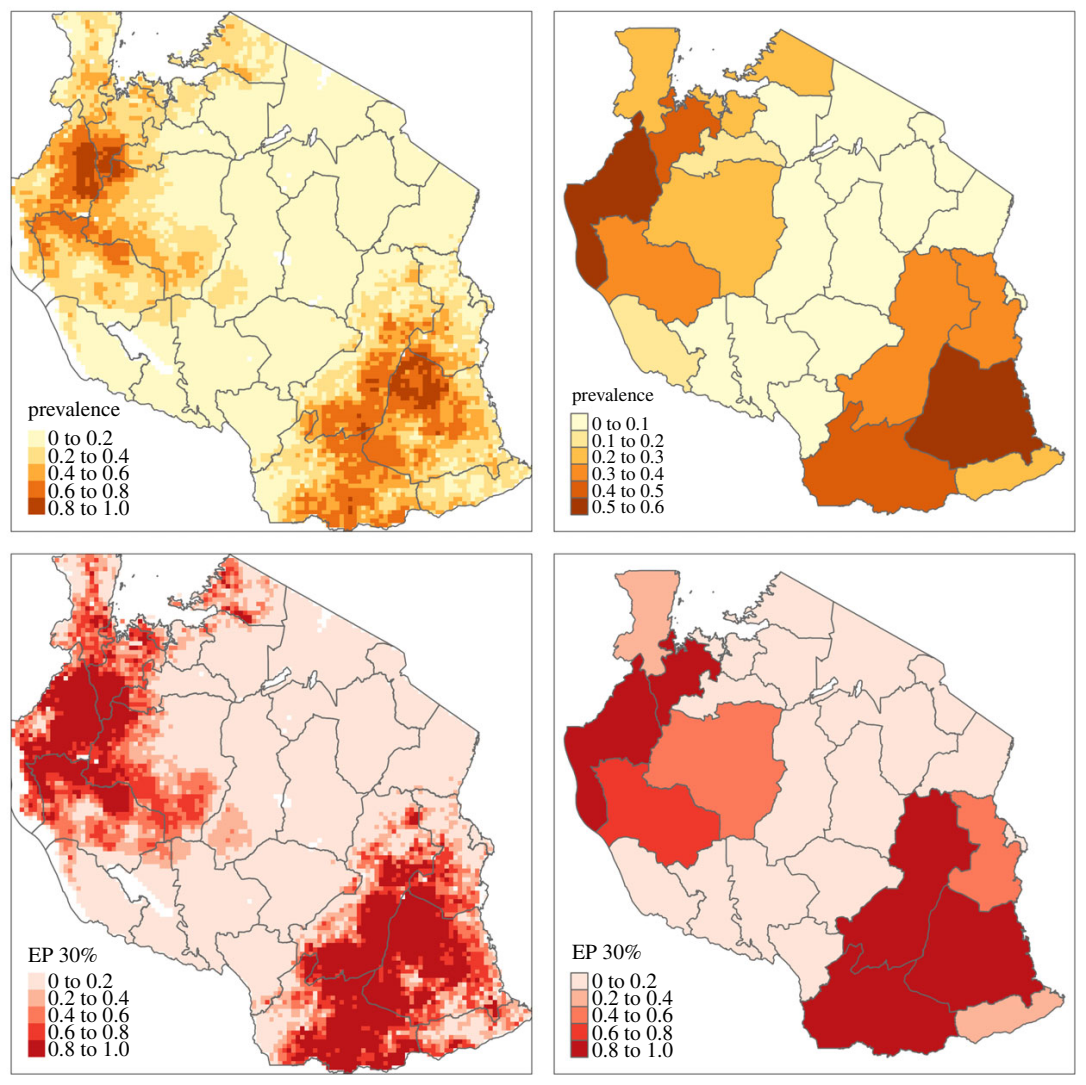
Left panels: maps of the predicted *P. falciparum* prevalence (upper panel) and of the exceedance probability for a 0.3 prevalence threshold (lower panel) on a 10 by 10 km regular grid. Right panels: maps of the predicted average prevalence, weighted by population density, at regional level (upper panel) and the probability that this exceeds a 0.3 prevalence threshold (lower panel).

Figure 4 shows the predicted prevalence and the EP for a 0.3 threshold over the regular grid (left panels) and at regional level (right panels). The regional-level estimates are obtained by using population density as the weighting function *w*(*x*). We identify areas that are highly likely to exceed 0.3 prevalence in the northwest and southeast of the country, where the EP is at least 80%. Large swathes of the central areas of Tanzania have instead estimated prevalences below 20% and EPs no more than 20%, indicating a low probability of exceeding a 0.3 threshold. The predictions at regional level mask the spatial variation observed over the grid locations. The largest estimated prevalence is about 0.47 in the North-Western region of Geita. In the lower right panel of figure 4, we see that overall three districts in Tanzania have an EP of at least 80% of a exceeding a 0.3 threshold.

## 6. Model validation

The next step of the geostatistical analysis concerns three aspects of the validation of a fitted geostatistical model: (a) compatibility of the chosen covariance function with the data; (b) calibration of the predictive distribution of prevalence; (c) validation of the assumption of conditional independence.

To pursue (a), we use a Monte Carlo procedure based on the empirical variogram. We first simulate a large number of binomial datasets under the fitted model, say 10 000. For each of these, we fit the model in (3.2) to obtain our estimates *Z*_*i*_ and the $v_{ij}={\left({\hat{Z}}_{i}-{\hat{Z}}_{\;j}\right)}^{2}/2$. The resulting 10 000 variograms are used to compute 95% probability intervals at each of the distance bins. If the variogram obtained from the original data falls within the resulting envelope we conclude that the chosen spatial correlation function gives a suitable model. Using this approach in our application, we conclude that the assumed exponential spatial correlation is supported by the data, as evidenced by electronic supplementary material 1, figure S4. Failure of this diagnostic check could indicate misspecification of the covariance function or incorrect omission of the nugget effect *Z*_*i*_ in (4.2). These and other examples are shown in §4.3 of Diggle & Giorgi [40]. Note, however, that the 95% probability intervals shown in electronic supplementary material 1, figure S4 are wide, indicating low statistical power to reject the model; in our experience, rejection of the correlation function based on the outlined Monte Carlo approach is more likely to occur at smaller distance bins of the variogram, where the test is more powerful.

For the assessment of the model calibration (b), we adapt the probability integral transform for count data proposed by Czado *et al.* [43] to binomial geostatistical models. The details and application of this approach are described in electronic supplementary material 1, §1. In brief, this consists of the following steps.

1. Randomly split the dataset into a training and test set.
2. Obtain the predictive distribution of prevalence at the locations of the test set, based on the fitted geostatistical models.
3. Apply the probability integral transform (defined in equation (3) of electronic supplementary material 1) to the observed positive counts of the test set.
4. Assess through the cumulative density function whether the transformed data from the previous step follow a uniform distribution.

One of the main advantages of this approach over cross-validated prediction error summaries, such as the mean square prediction error, is that it does not rely on treating the fraction of positive cases as if it were the true prevalence. Also, the use of the probability integral transform allows us to assess the overall compatibility of the predictive distribution of prevalence with the data, rather than just its mean. The results reported in electronic supplementary material do indicate that $M_{F}$ is a well calibrated model. For other approaches to the predictive assessment of count data, we refer the reader to Czado *et al.* [43].

Finally, for the third objective (c), we first compute Pearson’s residuals, defined as

$$e_{i}=\frac{\left(y_{i}-n_{i}\hat{\;p}(x_{i})\right)}{\sqrt{n_{i}\hat{\;p}(x_{i})(1-\hat{\;p}(x_{i}))}},$$

where $\hat{\;p}(x_{i})$ is the mean of the predictive distribution of prevalence at location *x*_*i*_, for *i* = 1, …, *n*. We then compute the variogram based on *e*_*i*_ and generate the 95% confidence envelope using the permutation test, described above. The results are displayed in electronic supplementary material, figure S6 and show no evidence of residual spatial correlation in the residuals *e*_*i*_, which thus support the assumption of conditional independence.

## 7. Assessment of the contribution of covariates to spatial prediction

We now assess the contribution of the domain effects, *δ*_*j*_(*x*_*i*_), to the prevalence predictions.

To this end, we first fit and carry out spatial prediction for the following four geostatistical models, each of which considers a subset of the covariates used in the full model.

— $M_{0}$: the model that excludes all covariates and relies exclusively on the spatial process *S*(*x*_*i*_) to interpolate *p*(*x*_*i*_).
— $M_{E}$: the model that only includes environmental variables, i.e. EVI, temperature and precipitation.
— $M_{\mathrm{SES}}$: the model that only includes socio-economic-status variables, i.e. population density and NTL.
— $M_{F}$: the full model.

The parameter estimates for each of these models are reported in electronic supplementary material, tables S2 to S5.

We then compare the models to assess how the introduction of covariates affects (i) the spatial structure of the unexplained variation in prevalence *S*(*x*_*i*_) + *Z*_*i*_, (ii) the point estimates of prevalence and (iii) the exceedance probabilities (EPs).

To carry out (i), we examine changes in the estimates of *σ*^2^, *ϕ* and *τ*^2^ through a graphical inspection of the theoretical variograms from the four fitted models. When using an exponential correlation function, as in our application, the theoretical variogram is

$$\tau^{2}+\sigma^{2}(1-\exp\{-u/\phi \}).$$

Plugging the maximum-likelihood estimates into the above equation, gives the four variograms shown in electronic supplementary material, figure S5. This clearly shows that the introduction of covariates leads to smaller estimates for *σ*^2^, larger values for *ϕ* but does not materially change the estimate of the nugget variance *τ*^2^. The practical range, i.e. the distance that gives a spatial correlation of 0.05, obtained from $M_{\mathrm{SES}}$ is closer to $M_{F}$ than to the other two models, suggesting that socio-economic variables explain spatial variation on a smaller scale than environmental covariates.

To quantify the impact of the covariates on the spatial predictions of the predictive target, we consider two summaries defined for both spatially continuous and spatially aggregated predictions.

The first summary quantifies how similar are the prevalence predictions from $M_{0}$, $M_{\mathrm{SES}}$ and $M_{E}$ compared with the full model $M_{F}$, using the root-mean-integrated-square-error (RMISE) criterion,

7.1 $${\mathrm{RMISE}}_{C}={\left(\frac{1}{\left|A\right|}\int_{A}{\left\{{\hat{\;p}}_{M_{i}}(x)-{\hat{\;p}}_{M_{F}}(x)\right\}}^{2}\;dx\right)}^{1/2},$$

where ${\hat{\;p}}_{M_{F}}(x)$ and ${\hat{\;p}}_{M_{i}}(x)$ are the point predictions of *p*(*x*) from $M_{F}$ and $M_{i}$, for *i* = 0, SES or *E*. Values of RMISE_*C*_ close to 0 indicate that model $M_{i}$ delivers predictions that are closer to the full model $M_{F}$. Similarly, for the spatially aggregated estimates *p*(*R*_*k*_) in (5.1), we compute

7.2 $${\mathrm{RMISE}}_{R}={\left(\frac{1}{K}\sum_{k=1}^{K}{\left\{{\hat{\;p}}_{M_{i}}(R_{k})-{\hat{\;p}}_{M_{F}}(R_{k})\right\}}^{2}\right)}^{1/2}$$

which we interpret in analogous fashion to RMISE_*C*_.

To assess the impact of the covariates on the EPs, we propose a summary index that quantifies how well a fitted model is able to discriminate between areas that are above and below a given threshold. Hence, the summary should penalize models that give EPs closer to 0.5 and favour models with EPs closer to 0 or 1. For the spatially continuous EPs, this can be achieved using the following index:

7.3 $$I_{C}=-\int_{A}{\left({\mathrm{EP}}_{M_{i}}(x)-0.5\right)}^{2}\;dx.$$

Similarly, for the EPs of the spatially aggregated average prevalence

7.4 $$I_{R}=-\sum_{k=1}^{K}{\left({\mathrm{EP}}_{M_{i}}(R_{k})-0.5\right)}^{2}.$$

As both *I*_*C*_ and *I*_*R*_ can only take negative values, these two indices are close to 0 when the EPs are close to 0.5. Conversely, *I*_*C*_ and *I*_*R*_ will give large negative values for EPs that are closer to 0 or 1.

In our application, we approximate the integrals in (7.1) and (7.3) using the 10 by 10 km regular grid shown in electronic supplementary material, figure S3. The values of the proposed summaries for each model are reported in table 2. Regarding the spatially continuous predictive inferences, we observe that $M_{\mathrm{SES}}$ gives prevalence predictions that are closer to $M_{F}$ than the other models. The same is also observed for the regional predictions of the average prevalence. The model that gives a better discrimination of areas above and below a 0.5 prevalence threshold is $M_{E}$ but, when considering regional-level EPs, all the models considered give similar performances.

**Table 2.** Summaries quantifying the contribution of the covariates to spatial prediction. The summaries RMISE_*C*_ and RMISE_*R*_ quantify the discrepancy of the point predictions of prevalence between $M_{0}$, $M_{E}$ and $M_{\mathrm{SES}}$ with the full model $M_{F}$. The summaries *I*_*C*_ and *I*_*R*_ quantify how close the exceedance probabilities generated from a model are to 0 or 1. The interpretation of the summaries is explained in the main text.

	model			
index	$M_{0}$	$M_{E}$	$M_{\mathrm{SES}}$	$M_{F}$
RMISE_*C*_	0.027	0.026	0.013	—
RMISE_*R*_	0.176	0.181	0.046	—
*I*_*C*_	−1397.012	−1491.385	−1394.219	−1443.877
*I*_*R*_	−6.002	−6.178	−5.754	−5.998

We conclude that, in this example, NTL and population density are mostly driving the predictions of prevalence in the final model, both on a continuous and regional scale. Environmental variables help to better discriminate between areas above and below a 0.3 prevalence threshold on a spatially continuous scale. However, according to the EPs of spatially aggregated prevalences at regional-level, the inclusion of covariates does not improve the discrimination between spatial units that exceed and do not exceed 0.3.

### 7.1. Further insights into the impact of covariates through a simulation study

We carry out a simulation study in order to gain further insights into the impact on spatial prediction arising from: (1) the misspecification of the relationship between covariates and prevalence; (2) the omission of important covariates.

In our simulations, we use $M_{I}$ as the true model for generating simulated datasets. We then compare the performance of the true model $M_{I}$ with $M_{F}$, which misspecifies the relationships with NTL and precipitation, and with a model that does not use any covariates, denoted by $M_{0}$; see table 3.

**Table 3.** Equations of the relationships between covariates and prevalence that are fitted in the simulation study under the true model ($M_{i}$) and the misspecified model ($M_{F}$). To keep the mathematical notation simple, each covariate is denoted identically as *d*(*x*) and regression coefficients are omitted in each equation. Note that *d*(*x*) + max{*d*(*x*) − *c*, 0} denotes a linear spline with a single change point at *d*(*x*) = *c*.

covariate	$M_{I}$	$M_{F}$
temperature	*d*(*x*)	*d*(*x*)
EVI	*d*(*x*) + max{*d*(*x*) − 0.35, 0}	*d*(*x*) + max{*d*(*x*) − 0.35, 0}
NTL	*d*(*x*) + max{*d*(*x*) − 9, 0}	*d*(*x*)
log-population	*d*(*x*)	*d*(*x*)
log-precipitation	*d*(*x*) + max{*d*(*x*) − 6.85, 0}	*d*(*x*)

The simulation proceeds through the following iterative steps.

1. Simulate the true prevalence under $M_{I}$ at the observed locations (figure 1) and over the 10 by 10 km regular grid (electronic supplementary material 1, figure S3).
2. Based on the simulated prevalence at observed locations in the previous step, simulate a binomial dataset for the number of malaria cases.
3. Fit models $M_{I}$, $M_{F}$ and $M_{0}$ to the simulated dataset in the previous step.
4. For each of the three fitted models, carry out spatial predictions for prevalence over the 10 by 10 km regular grid and at regional level.
5. Repeat steps 1 to 4, 1000 times.

On completion of these steps, we summarize the performance of $M_{I}$, $M_{F}$ and $M_{0}$, through summaries at both pixel-level and regional-level. We quantify the prediction error from the three models using the RMISE as defined in the previous section, replacing ${\hat{\;p}}_{M_{I}}(x)$ in (7.1) and ${\hat{\;p}}_{M_{I}}(R_{k})$ in (7.2) with the true prevalences obtained in step 1. We then denote these two summaries as ${\mathrm{RMISE}}_{C}^{T}$ and ${\mathrm{RMISE}}_{R}^{T}$, respectively. To quantify the accuracy of the classifications of both pixels and regions based on the EPs, we first optimize the sensitivity and specificity of each model and average these two across the 1000 simulations, as follows. For each simulated dataset, we use the simulated true prevalence, both at pixel-level and regional-level, to label each pixel and region as being above or below the threshold of 0.3 prevalence. Based on the fitted geostatistical model, we then compute the EPs of 0.3 prevalence and find the value of EP that gives the largest sensitivity and specificity for the classification of pixels and regions. In reporting the results, we use Sens_*C*_ and Spec_*C*_ denote the sensitivity and specificity, respectively, at pixel-level, and Sens_*R*_ and Spec_*R*_ similarly at regional-level.

The results of the simulation study are reported in table 4. The true model $M_{I}$ yields the smallest RMISE for both the regional and spatially continuous predictions. The predictive performance of the misspecified model $M_{F}$ in terms of RMISE is not materially different from that of the true model for both continuous and regional targets. However, the misspecified relationship leads to a substantial decrease in the sensitivity of the model to detect areas above a 0.3 prevalence. The model without covariates, $M_{0}$, has a higher RMISE than the other two models but also a substantially higher sensitivity than *M*_*F*_. This illustrates the general point that model choice needs to be considered in relation to the purpose of the modelling.

**Table 4.** Results of the simulation study for the true model $M_{I}$, the misspecified model $M_{F}$ and the model excluding all the covariates $M_{0}$. The covariates used in $M_{I}$ and $M_{F}$, and their relationship with prevalence are defined in table 3. For a definition of the summaries, see the main text.

	model		
summary	$M_{I}$	$M_{F}$	$M_{0}$
${\mathrm{RMISE}}_{C}^{T}$	0.105	0.129	0.151
Spec_*C*_	0.890	0.929	0.899
Sens_*C*_	0.936	0.762	0.927
${\mathrm{RMISE}}_{R}^{T}$	0.034	0.039	0.056
Spec_*R*_	0.977	0.998	0.899
Sens_*R*_	0.989	0.585	0.717

## 8. Discussion

In this paper, we have introduced a framework for the geostatistical analysis of spatially referenced prevalence data and have illustrated the different stages of the analysis using a case study on malaria mapping in Tanzania. The aim of this framework is to guide researchers to make modelling choices that enhance the predictive or explanatory power of the model depending on the primary objective of the analysis. For each step of a geostatistical analysis, we have proposed the use of statistical tools to address specific modelling objectives. We emphasize that the list of statistical tools presented in table 1 is not exhaustive and alternative solutions are also possible. Below, we further consider the strengths and limitations of our proposed methods.

The models presented in this paper did not explicitly account for the stratification of the DHS-MIS2015 conducted in Tanzania. In electronic supplementary material 2, we revisit the final model, namely $M_{F}$, and incorporate the strata variables provided by the DHS-MIS2015, corresponding to the urban and rural strata for each of the regions of Tanzania. The results suggest that the inclusion of population density, NTL and the spatial random effects *S*(*x*) adequately adjust for the urban/rural stratification, thus making the inclusion of the design-based variables unnecessary. However, we emphasize that this result cannot be generalized and the importance of stratification variables should be assessed in any geostatistical analysis. As explained in §2 of Giorgi *et al.* [41], failing to account for the stratification of the design can lead to a stochastic dependence between the sampling design and the underlying spatial process—also known as *preferential sampling*—which can invalidate the predictive inference on prevalence. In the context of vaccination coverage estimation, Dong & Wakefield [44] have carried out a thorough study on the importance of design variables into geostatistical models, showing that modelling survey stratification and clustering improves the predictive performance of geostatistical models. Previous studies that used geostatistical models for disease mapping did not account for the sampling design explicitly (e.g. [45–48]), but made extensive use of covariates that are correlated with the urban and rural strata and may therefore be sufficient to adjust for the stratification of the design.

The problem of accounting for the sampling design has been extensively investigated in the field of *small area estimation*, or SME (e.g. [49–51]). SME models are spatially discrete models that are used to analyse areal-level outcomes and borrow strength of information across space by defining a spatial correlation structure based on neighbouring properties. SME methods thus provide an alternative approach to model-based geostatistical methods when the goal of the analysis is exclusively focused on making predictive inferences on disease risk at district-level. The recent study by Utazi *et al.* [52] compared the predictive performances of SME and model-based geostatistical models, and found that the latter deliver equally reliable areal-level estimates of prevalence. However, as highlighted by Paige *et al.* [53], district-level estimates based on geostatistical models require the availability of high-quality population density surfaces for the computation of areal-level prevalences (see equation (5.1)) and should, in principle, also be stratified according to the design. Nevertheless, model-based geostatistical methods provide principled, likelihood-based solutions to questions that cannot be addressed with SME methods, including the ability to combine data at multiple spatial resolutions and to carry out spatial aggregation at whatever level is relevant to a given health policy question.

Another important topic that we did not cover in this paper is the handling of large spatial datasets, which can substantially increase the computational burden. For an overview of some of the current methodologies that are used to approximate a Gaussian process, we refer the reader to ch. 3 of Diggle & Giorgi [40]. Among these, the approximation based on stochastic partial differential equations (SPDE), proposed by Lindgren *et al.* [54], is one of the most commonly used, especially in conjunction with Bayesian inference [55].

In order to guide the model-building process, we have distinguished between the predictive and explanatory power of the model. The balance between the two should depend on the objectives of the analysis. In our data example on malaria mapping in Tanzania, the objective was to identify areas of high transmission, defined as exceedance of a 0.3 prevalence threshold. Among the models considered, $M_{I}$, which included both environmental and socio-economic risk factors as covariates, had the strongest explanatory power. However, our results indicated that some of the regression coefficients of the linear splines were poorly identified and proposed a simplified model, *M*_*F*_, which incorporated most covariates as linear effects into the linear predictor. From a predictive modelling perspective, when considering models that use subsets of the covariates in *M*_*F*_, we found that the full model $M_{F}$ delivered only a marginally better performance than those. Also, we found that some covariates helped to improve the accuracy of the point predictions of prevalence but were not as useful in increasing the sensitivity and specificity of the model with respect to the identification of pixels and regions that exceeded a 0.3 prevalence threshold. Furthermore, our simulation study showed that a model that misspecified the relationship between the covariates and prevalence performed as well as the true model in terms of the root-mean-square-error, but had a substantially lower sensitivity in the identification of areas above 0.3 prevalence. We point out that these findings are dependent on both the properties of the underlying spatial process and the prevalence threshold used for the exceedance probabilities, hence they are not generalizable to other geostatistical analyses.

We have shown that linear splines can help to account for non-linear relationships between covariates and disease prevalence, and to explain covariate effects through easily interpreted regression coefficients. We have also introduced the concept of domain effects in order to inform the variable selection process and the validation of the model. When considering a larger number of covariates than those used in our example, a reduction of the dimensionality could be achieved by combining all the covariates within each domain using, for example, principal component analysis. However, in this case, the explanatory power of the model would rely on interpretability of the principal components, which may be problematic.

In our framework, validation of the assumed correlation function is carried out through a Monte Carlo procedure based on the empirical variogram. Two limitations of this approach are: the validity of the diagnostic relies on the assumption of stationary and isotropy; the statistical power to reject a chosen correlation function is often low. The second issue is related to the wider problem that prevalence data are only weakly informative of the smoothness of the underlying spatial process, which can result in highly uncertain estimates of the covariance parameters. Development of more statistically efficient tools for the diagnostic of correlation functions for count data requires more research.

To assess over-fitting, we have proposed the use of the correlation matrix as an approach for exploring overfitting and identify solutions for avoiding over-complex and poorly estimated regressions relationships. We emphasize that this approach cannot be used to assess the relative importance of covariates but only how well the regression relationships of the model are estimated. In our analysis of the Tanzania malaria data our initial geostatistical model did show evidence of overfitting with correlations close 1 and we therefore simplified the model by removing knots of the linear splines at points where the data were sparse. However, it is difficult to provide a single, universal threshold value for the correlation that can be applied to identify overfitting. Our view is that decisions on how to define regression relationships should also rely on subject matter knowledge and not exclusively on statistical judgement. For this reason, models that have more scientific validity but are less precisely estimated may also be favoured in some contexts.

In our example, information on the minimum and maximum age of the sampled individuals at each location was also available. However, we did not find any discernible relationship of these with the empirical prevalence and therefore discarded them from the analysis. When analysing aggregated disease counts data, an alternative approach to the standardization of prevalence maps to specific age groups is to define weights *w*(*x*) in (5.1) based on the age-stratified population density at location *x*.

In studies where the primary goal is to understand the relationship between risk factors and disease risk, rather than geostatistical prediction, some modifications to the framework we have illustrated may be needed. For example, a model for disease prevalence may be first developed by considering only covariates that are known to confound the relationship between disease risk and the variables that are of primary scientific interest. These are then introduced in the model after restricting the variable selection process, as described in this paper, to the confounding factors. Covariates of scientific interest should then be kept in the final model, regardless of their statistical significance. Confidence intervals for the estimated regression relations can instead be used as a way of conveying the strength of the association between the covariates and disease risk.

Finally, we point out that the statistical ideas and principles presented in this paper are applicable to any statistical analysis of epidemiological data based on regression modelling. This also includes extensions of the standard geostatistical model for prevalence mapping to spatio-temporal analysis, modelling of zero-inflated prevalence data, combining data from a mix of randomized and opportunistic surveys [56,57] and multiple diagnostics [58].

## Data accessibility

This article has no additional data.

## References

[1] Diggle PJ, Tawn JA, Moyeed RA. 1998 Model-based geostatistics (with discussion). Appl. Stat. **47**, 299-350.

[2] Shmueli G. 2010 To explain or to predict? Stat. Sci. **25**, 289-310. (10.1214/10-STS330)

[3] Brousse O, Georganos S, Demuzere M, Dujardin S, Lennert M, Linard C, Snow RW, Thiery W, Van Lipzig NP. 2015 Re-examining environmental correlates of *Plasmodium falciparum* malaria endemicity: a data-intensive variable selection approach. Malar. J. **14**, 68. (10.1186/s12936-015-0574-x) 25890035 PMC4333887

[4] Bhatt S, Cameron E, Flaxman SR, Weiss DJ, Smith DL, Gething PW. 2017 Improved prediction accuracy for disease risk mapping using Gaussian process stacked generalization. J. R. Soc. Interface **14**, 20170520. (10.1098/rsif.2017.0520) 28931634 PMC5636278

[5] George EI, McCulloch RE. 1993 Variable selection via gibbs sampling. J. Am. Stat. Assoc. **88**, 881-889. (10.1080/01621459.1993.10476353)

[6] Diboulo E, Sié A, Diadier DA, Voules DAK, Yé Y, Vounatsou P. 2015 Bayesian variable selection in modelling geographical heterogeneity in malaria transmission from sparse data: an application to Nouna Health and Demographic Surveillance System (HDSS) data, Burkina Faso. Parasites Vectors **8**, 118. (10.1186/s13071-015-0679-7) 25888970 PMC4365550

[7] Giardina F, Gosoniu L, Konate L, Diouf MB, Perry R, Gaye O, Faye O, Vounatsou P. 2012 Estimating the burden of malaria in Senegal: Bayesian zero-inflated binomial geostatistical modeling of the MIS 2008 data. PLoS ONE **7**, 1-10. (10.1371/annotation/e7549f68-308c-45d5-a14d-8b642a930495) PMC3293829 22403684

[8] Adigun AB, Gajere EN, Oresanya O, Vounatsou P. 2015 Malaria risk in Nigeria: Bayesian geostatistical modelling of 2010 malaria indicator survey data. Malar. J. **14**, 156. (10.1186/s12936-015-0683-6) 25880096 PMC4404580

[9] Chammartin F, H’urlimann E, Raso G, N’Goran EK, Utzinger J, Vounatsou P. 2013 Statistical methodological issues in mapping historical schistosomiasis survey data. Acta Trop. **128**, 345-352. (10.1016/j.actatropica.2013.04.012) 23648217

[10] Macharia PM, Giorgi E, Noor AM, Waqo E, Kiptui R, Okiro EA, Snow RW. 2018 Spatio-temporal analysis of *Plasmodium falciparum* prevalence to understand the past and chart the future of malaria control in Kenya. Malar. J. **17**, 340. (10.1186/s12936-018-2489-9) 30257697 PMC6158896

[11] Giorgi E, Osman AA, Hassan AH, Ali AA, Ibrahim F, Amran JG, Noor AM, Snow RW. 2018 Using non-exceedance probabilities of policy-relevant malaria prevalence thresholds to identify areas of low transmission in Somalia. Malar. J. **17**, 88. (10.1186/s12936-018-2238-0) 29463264 PMC5819647

[12] Slater H, Michael E. 2013 Mapping, bayesian geostatistical analysis and spatial prediction of lymphatic filariasis prevalence in africa. PLoS ONE **8**, 1-14. (10.1371/journal.pone.0071574) PMC3741112 23951194

[13] Moraga P *et al.* 2015 Modelling the distribution and transmission intensity of lymphatic filariasis in sub-Saharan Africa prior to scaling up interventions: integrated use of geostatistical and mathematical modelling. Parasites Vectors **8**, 560. (10.1186/s13071-015-1166-x) 26496983 PMC4620019

[14] Zouré HG, Noma M, Tekle AH, Amazigo UV, Diggle PJ, Giorgi E, Remme JH. 2014 The geographic distribution of onchocerciasis in the 20 participating countries of the African Programme for Onchocerciasis Control: (2) pre-control endemicity levels and estimated number infected. Parasites Vectors **7**, 326. (10.1186/1756-3305-7-326) 25053392 PMC4222889

[15] O’Hanlon SJ *et al.* 2016 Model-based geostatistical mapping of the prevalence of *Onchocerca volvulus* in West Africa. PLOS Negl. Trop. Dis. **10**, 1-36. (10.1371/journal.pntd.0004328) PMC4714852 26771545

[16] Magalhães R, Clements A, Patil A, Gething P, Brooker S. 2011 The applications of model-based geostatistics in helminth epidemiology and control. Adv. Parasitol. **74**, 267-296. (10.1016/b978-0-12-385897-9.00005-7) 21295680 PMC3037997

[17] Lai Y, Zhou X, Utzinger J, Vounatsou P. 2013 Bayesian geostatistical modelling of soil-transmitted helminth survey data in the People’s Republic of China. Parasites Vectors **6**, 359. (10.1186/1756-3305-6-359) 24350825 PMC3892068

[18] Ministry of Health, Community Development, Gender, Elderly and Children (MoHCDGEC), National Bureau of Statistics (NBS), Office of the Chief Government Statistician (OCGS), and ICF. Tanzania Demographic and Health Survey and Malaria Indicator Survey (TDHS-MIS) 2015-16. 2016. dhsprogram.com/pubs/pdf/fr321/fr321.pdf.

[19] Thawer S *et al.* 2020 Sub-national stratification of malaria risk in mainland Tanzania: a simplified assembly of survey and routine data. Malar. J. **19**, 1-12. (10.1186/s12936-020-03250-4) 32384923 PMC7206674

[20] Canelas T, Castillo-Salgado C, Ribeiro H. 2016 Systematized literature review on spatial analysis of environmental risk factors of malaria transmission. Adv. Infect. Dis. **6**, 52-62. (10.4236/aid.2016.62008)

[21] Dlamini SN, Beloconi A, Mabaso S, Vounatsou P, Impouma B, Fall IS. 2019 Review of remotely sensed data products for disease mapping and epidemiology. Remote Sensing Appl. Soc. Environ. **14**, 108-118. (10.1016/j.rsase.2019.02.005)

[22] Odhiambo JN, Kalinda C, Macharia PM, Snow RW, Sartorius B. 2020 Spatial and spatio-temporal methods for mapping malaria risk: a systematic review. BMJ Global Health **5**, e002919. (10.1136/bmjgh-2020-002919) PMC7537142 33023880

[23] Proville J, Zavala-Araiza D, Wagner G. 2017 Night-time lights: a global, long term look at links to socio-economic trends. PLoS ONE **12**, 1-12. (10.1371/journal.pone.0174610) PMC5367807 28346500

[24] Omumbo JA, Guerra C, Hay S, Snow R. 2005 The influence of urbanisation on measures of *Plasmodium falciparum* infection prevalence in East Africa. Acta Trop. **93**, 11-21. (10.1016/j.actatropica.2004.08.010) 15589793 PMC3191363

[25] Kabaria C, Gilbert M, Noor A, Snow R, Linard C. 2017 The impact of urbanization and population density on childhood *Plasmodium falciparum* parasite prevalence rates in Africa. Malar. J. **16**, 1-10. (10.1186/s12936-017-1694-2) 28125996 PMC5270336

[26] Funk C *et al.* 2015 The climate hazards infrared precipitation with stations—a new environmental record for monitoring extremes. Sci. Data **2**, 150066. (10.1038/sdata.2015.66) 26646728 PMC4672685

[27] Snow RW *et al.* 2017 The prevalence of *Plasmodium falciparum* in sub-Saharan Africa since 1900. Nature **550**, 515-518. (10.1038/nature24059) 29019978 PMC5660624

[28] Stanton MC, Diggle PJ. 2013 Geostatistical analysis of binomial data: generalised linear or transformed Gaussian modelling? Environmetrics **24**, 158-171. (10.1002/env.2205)

[29] Wood SN 2017 Generalized additive models: an introduction with R, 2nd edn. Chapman & Hall/CRC Texts in Statistical Science. New York, NY: CRC Press.

[30] Molineaux L. 1988 The epidemiology of human malaria as an explanation of its distribution, including some implications for its control. In *Malaria: principles and practice of malariology* (eds W Wernsdorfer, I McGregor), pp. 913–998. London, UK: Churchill Livingstone.

[31] Christiansen-Jucht CD, Parham PE, Saddler A, Koella JC, Basáñez MG. 2015 Larval and adult environmental temperatures influence the adult reproductive traits of *Anopheles gambiae* s.s. Parasites Vectors **8**, 456. (10.1186/s13071-015-1053-5) 26382035 PMC4573685

[32] Matérn B. 1986 Spatial variation. New York, NY: Springer.

[33] Giorgi E, Diggle P. 2017 PrevMap: an R package for prevalence mapping. J. Stat. Softw. **78**, 1-29. (10.18637/jss.v078.i08)

[34] Geyer CJ, Thompson EA. 1992 Constrained Monte Carlo maximum likelihood for dependent data. J. R. Stat. Soc. B **54**, 657-699.

[35] Geyer CJ. 1994 On the Convergence of Monte Carlo maximum likelihood calculations. J. R. Stat. Soc. B **56**, 261-274.

[36] Geyer CJ. 1996 Estimation and optimization of functions. In *Markov chain Monte Carlo in practice* (eds W Gilks, S Richardson, D Spiegelhalter), pp. 241–258. London, UK: Chapman and Hall.

[37] Christensen OF. 2004 Monte Carlo maximum likelihood in model-based geostatistics. J. Comput. Graph. Stat. **13**, 702-718. (10.1198/106186004X2525)

[38] Simpson D, Rue H, Riebler A, Martins TG, Sørbye SH. 2017 Penalising model component complexity: a principled, practical approach to constructing priors. Stat. Sci. **32**, 1-28. (10.1214/16-sts576)

[39] Fuglstad GA, Simpson D, Lindgren F, Rue H. 2019 Constructing priors that penalize the complexity of gaussian random fields. J. Am. Stat. Assoc. **114**, 445-452. (10.1080/01621459.2017.1415907)

[40] Diggle P, Giorgi E. 2019 Model-based geostatistics for global public health: methods and applications. Boca Raton, FL: Chapman and Hall/CRC Press.

[41] Giorgi E, Diggle PJ, Snow RW, Noor AM. 2018 Geostatistical methods for disease mapping and visualisation using data from spatio-temporally referenced prevalence surveys. Int. Stat. Rev. **86**, 571-597. (10.1111/insr.12268) 33184527 PMC7116348

[42] Hastie T, Tibshirani R, Friedman J 2001 The elements of statistical learning. Springer Series in Statistics. New York, NY: Springer.

[43] Czado C, Gneiting T, Held L. 2009 Predictive model assessment for count data. Biometrics **65**, 1254-1261. (10.1111/j.1541-0420.2009.01191.x) 19432783

[44] Dong TQ, Wakefield J. 2021 Modeling and presentation of vaccination coverage estimates using data from household surveys. Vaccine **39**, 2584-2594. (10.1016/j.vaccine.2021.03.007) 33824039 PMC9384691

[45] Gething P, Tatem A, Bird T, Burgert-Brucker CR. 2015 Creating spatial interpolation surfaces with DHS data. DHS Spatial Analysis Reports no11. Rockville, MD: ICF International.

[46] Gething PW *et al.* 2016 Mapping *Plasmodium falciparum* mortality in Africa between 1990 and 2015. N Engl. J. Med. **375**, 2435-2445. (10.1056/NEJMoa1606701) 27723434 PMC5484406

[47] Osgood-Zimmerman A *et al.* 2018 Mapping child growth failure in Africa between 2000 and 2015. Nature **555**, 41-47. (10.1038/nature25760) 29493591 PMC6346257

[48] Utazi CE, Thorley J, Alegana VA, Ferrari MJ, Takahashi S, Metcalf CJ, Lessler J, Tatem AJ. 2018 High resolution age-structured mapping of childhood vaccination coverage in low and middle income countries. Vaccine **36**, 1583-1591. (10.1016/j.vaccine.2018.02.020) 29454519 PMC6344781

[49] Chen C, Wakefield J, Lumely T. 2014 The use of sampling weights in Bayesian hierarchical models for small area estimation. Spatial Spatio-temporal Epidemiol. **11**, 33-43. (10.1016/j.sste.2014.07.002) PMC4357363 25457595

[50] Mercer L, Wakefield J, Chen C, Lumley T. 2014 A comparison of spatial smoothing methods for small area estimation with sampling weights. Spatial Stat. **8**, 69-85. (doi:Spatial Statistics Miami) 10.1016/j.spasta.2013.12.001 PMC4064473 24959396

[51] Watjou K, Faes C, Lawson A, Kirby RS, Aregay M, Carroll R, Vandendijck Y. 2017 Spatial small area smoothing models for handling survey data with non-response. Stat. Med. **36**, 3708-3745. (10.1002/sim.7369) 28670709 PMC5585068

[52] Utazi CE, Nilsen K, Pannell O, Dotse-Gborgbortsi W, Tatem AJ. 2021 District-level estimation of vaccination coverage: discrete vs continuous spatial models. Stat. Med. **40**, 2197-2211. (10.1002/sim.8897) 33540473 PMC8638675

[53] Paige J, Fuglstad GA, Riebler A, Wakefield J. 2020 Design- and model-based approaches to small-area estimation in a low- and middle-income country context: comparisons and recommendations. J. Survey Stat. Methodol. smaa011. (10.1093/jssam/smaa011)

[54] Lindgren F, Rue H, Lindström J. 2011 An explicit link between Gaussian fields and Gaussian Markov random fields: the stochastic partial differential equation approach. J. R. Stat. Soc. B (Stat. Methodol.) **73**, 423-498. (10.1111/j.1467-9868.2011.00777.x)

[55] Rue H, Martino S, Chopin N. 2009 Approximate Bayesian inference for latent Gaussian models by using integrated nested Laplace approximations. J. R. Stat. Soc. B (Stat. Methodol.) **71**, 319-392. (10.1111/j.1467-9868.2008.00700.x)

[56] Diggle PJ, Giorgi E. 2016 Model-based geostatistics for prevalence mapping in low-resource settings. J. Am. Stat. Assoc. **111**, 1096-1120. (10.1080/01621459.2015.1123158)

[57] Giorgi E, Sesay SS, Terlouw DJ, Diggle PJ. 2015 Combining data from multiple spatially referenced prevalence surveys using generalized linear geostatistical models. J. R. Stat. Soc. A (Stat. Soc.) **178**, 445-464. (10.1111/rssa.12069)

[58] Amoah B, Diggle PJ, Giorgi E. 2020 A geostatistical framework for combining spatially referenced disease prevalence data from multiple diagnostics. Biometrics **76**, 158-170. (10.1111/biom.13142) 31449327

